# How the wisdom of crowds, and of the crowd within, are affected by expertise

**DOI:** 10.1186/s41235-021-00273-6

**Published:** 2021-02-05

**Authors:** Joshua L. Fiechter, Nate Kornell

**Affiliations:** Department of Psychology, Williams College, 25 Stetson Ct., Williamstown, MA 01267 USA

## Abstract

We investigated the effect of expertise on the wisdom of crowds. Participants completed 60 trials of a numerical estimation task, during which they saw 50–100 asterisks and were asked to estimate how many stars they had just seen. Experiment 1 established that both inner- and outer-crowd wisdom extended to our novel task: Single responses alone were less accurate than responses aggregated across a single participant (showing inner-crowd wisdom) and responses aggregated across different participants were even more accurate (showing outer-crowd wisdom). In Experiment 2, prior to beginning the critical trials, participants did 12 practice trials with feedback, which greatly increased their accuracy. There was a benefit of outer-crowd wisdom relative to a single estimate. There was no inner-crowd wisdom effect, however; with high accuracy came highly restricted variance, and aggregating insufficiently varying responses is not beneficial. Our data suggest that experts give almost the same answer every time they are asked and so they should consult the outer crowd rather than solicit multiple estimates from themselves.

The average value of multiple estimates tends to be more accurate than any one single estimate; this phenomenon is known as the *wisdom of the crowd* (Surowiecki 2004). Galton (1907) published the first demonstration of the wisdom of the crowd. He analyzed responses from a weight-estimation game wherein people were trying to estimate the weight of an ox “after being slaughtered and dressed.” The mean estimate of all participants was 1197 lb; a re-analysis of Galton's notes showed that the correct weight of the ox was 1197 lb, meaning the crowd had perfectly assessed the weight (Wallis 2014).

Subsequent work has extended wisdom of the crowd to geopolitical forecasts (Mellers et al. 2014, 2016, 2017; Turner et al. 2014), probability estimates (Ariely et al. 2000; Lee and Danileiko 2014), ordering problems (e.g., the order of U.S. Presidents; Steyvers et al. 2009), forced-choice questions (Bennett et al. 2018), and tasks involving the coordination of multiple pieces of information, such as picking the most efficient path through a predetermined ordering of points (Yi et al. 2012). Furthermore, crowd wisdom has been observed in populations whose cognitive abilities are more limited than those of human adults, including young adolescents (Ioannou et al. 2018) and nonhuman animals (Ioannou 2017).

Remarkably, the benefits of averaging estimates hold even when those estimates come from the same person; this effect is called the *wisdom of the inner crowd* (see Herzog and Hertwig 2014a, for a review; see Ariely et al. 2000, for boundary conditions on the inner crowd). For example, Vul and Pashler (2008) asked participants eight general knowledge questions, all of which required an estimate of a percentage (e.g., What percentage of the world's airports are in the United States?). Participants were then unexpectedly asked all eight questions again, either immediately or three weeks later. The average of both guesses was more accurate than either the first or second guess alone, especially for the participants who waited three weeks between guesses.

The wisdom of the inner crowd has been observed with percentage estimation (Fraundorf and Benjamin 2014; Herzog and Hertwig 2014b; Hourihan and Benjamin 2010; Müller-Trede 2011; Steegen et al. 2014), numerical general knowledge estimation (Rauhut and Lorenz 2011; but see Müller-Trede 2011), date estimation (Herzog and Hertwig 2009; Müller-Trede 2011), and quantity estimation (i.e., guessing the number of objects in a container; van Dolder and van den Assem 2017). The benefits of delaying a subsequent guess have also replicated (Steegen et al. 2014; van Dolder and van den Assem 2017).

## Crowd variance and crowd wisdom

Following previous studies (e.g., Page 2007; Rauhut and Lorenz 2011; van Dolder and van den Assem 2017) we will focus on three derived values to assess crowd wisdom: (1) bias, or the squared distance from the crowd’s mean to the true value; (2) mean squared error (MSE), or the average squared distance from each estimate and the true value; and (3) variance, or the average squared distance from each estimate and the crowd’s mean (see Table 1 for an example of how these values are calculated). Bias indicates the error of a crowd and MSE indicates the error of an average individual estimate; thus, crowd wisdom can be defined as MSE − bias.^1^ Page (2007) demonstrated that variance = MSE − bias. He called this fact the *diversity prediction theorem*: the wisdom of a crowd is determined by the variance of its responses.

**Table 1:** Example of two crowds, each comprised of three estimates

Estimate #	Error	Squared error	Squared deviation from average
A			
1	12	144	136.11
2	− 14	196	205.44
3	3	9	7.11
	Bias	MSE	Variance
	0.11	116.33	116.22
B			
1	0	0	0.11
2	− 2	4	2.78
3	1	1	1.78
	Bias	MSE	Variance
	0.11	1.67	1.56

The true value being estimated is 0 (i.e., estimates and errors are equivalent). The two crowds consist of more (a) and less (b) estimate variance. Crowd wisdom (MSE–bias) is equal to the variance of the estimates of the crowd. Crowd wisdom is tautologically less advantageous when variance is small (b)

Bias is the mean of the "Error" column, squared. MSE is the mean of the "Squared error" column. Variance is the mean of the "Squared deviation from average" column

The diversity prediction theorem (Page 2007) provides a convenient conceptualization of the findings discussed so far. First, inner- and outer-crowd wisdom will be evident so long as estimates vary to a sufficiently large degree. Second, the benefit of spacing estimates from the inner crowd (e.g., Vul and Pashler 2008) arises from the fact that estimates will be less correlated, and therefore more varied, when more time has passed between those estimates. We tested an additional implication of the diversity prediction theorem that has received no previous empirical testing (but see Hong and Page (2004), for relevant simulations): crowd wisdom might suffer under conditions in which people have expertise. The reasoning behind this claim is that experts may tend to rely on the same information, either between or within individuals, and therefore will produce an insufficiently varied set of estimates.

The present experiments evaluated expertise and the wisdom of the inner and outer crowds in a novel numerosity estimation task (adapted from Kornell and Hausman 2017). We chose this task for multiple reasons: First, people tend to produce inaccurate estimates in such tasks (Minturn and Reese 1951), primarily underestimating the number of items displayed (Indow and Ida 1977; Izard and Dehaene 2008; Krueger 1982, 1984); second, people can be quickly trained to calibrate their estimates (Izard and Dehaene 2008; Krueger 1984; Lipton and Spelke 2005); third, regarding the inner crowd, it is possible to ask the same question multiple times without a long delay by showing the same number of items but arranging them in different configurations. We hoped that these properties would enhance our prospects of observing the effect of expertise on the inner and outer crowd. In Experiment 1 we evaluated whether the wisdom of the inner and outer crowd extended to our novel task. In Experiment 2 we asked whether crowd wisdom persisted after we made our participants experts at the task via training trials.

## Experiment 1

The goal of Experiment 1 was to replicate the wisdom of the inner and outer crowd in our novel numerosity estimation task.

### Methods

#### Participants

Participants were 63 people recruited from Amazon's Mechanical Turk service. All participants were paid $1.00 to complete the experiment; pay did not reflect performance on the task. Previous attempts at replicating laboratory findings on Mechanical Turk have generally been successful (e.g., Crump et al. 2013) and so we felt that our participants would be motivated to perform well even with a flat pay rate. We collected data from 70 people, anticipating that we would obtain usable data from approximately 60 of them.^2^ We did not analyze data from participants who began the experiment multiple times, did not report being fluent in English, reported experiencing technical difficulties, or reported having seen our stimuli before.

#### Procedure

**Fig. 1:**
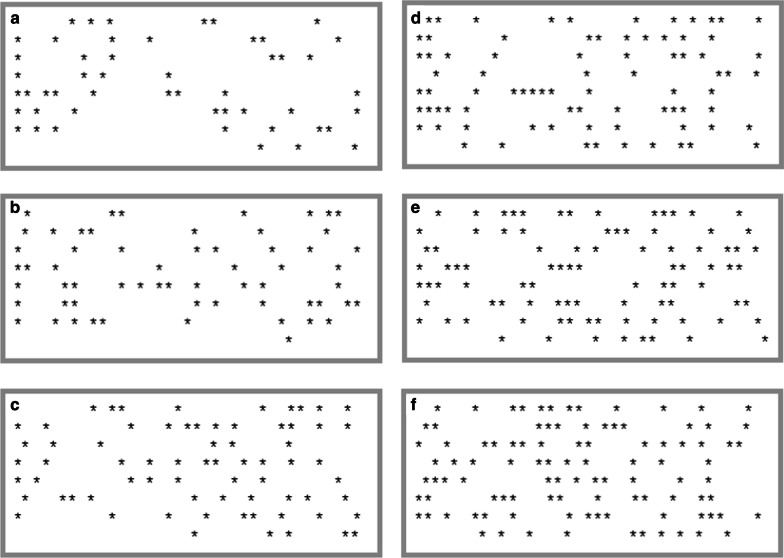
Example displays of each set size: 50 (**a**), 60 (**b**), 70 (**c**), 80 (**d**), 90 (**e**), and 100 (**f**) stars

Participants viewed a box containing asterisks (*) on a computer screen (see Fig. 1). They completed 60 trials in total, ten trials each of six different set sizes (50, 60, 70, 80, 90, or 100 stars). The order of these 60 trials was randomly determined for each participant and the positioning of the stars was randomly determined on each trial. Participants viewed the star-filled box for 2 s; the box was then removed from the screen and participants were asked to estimate the number of stars present in the box.

#### Dependent variable

Response accuracy is typically measured using mean squared error (i.e., MSE) and squared error (i.e., bias). In this study, however, errors tended to be larger for larger set sizes. To eliminate this noise we converted the error of the estimates into a proportion of the true value being estimated (Rauhut and Lorenz 2011; van Dolder and van den Assem 2017). For example, a response of 55 or 45 when there were 50 stars was coded as 0.10 or − 0.10, respectively. We calculated our dependent variable, mean squared proportional-error (MSE_P_),^3^ based on these proportions. The MSE_P_ would be 0.01 for both 0.1 and − 0.1. We did not log-transform participants' responses, as previous studies have done with numerical estimates (Rauhut and Lorenz 2011; van Dolder and van den Assem 2017). This decision arose from the fact that participants' responses were skewed in Experiment 1 but not in Experiment 2; we therefore elected to not transform responses for either experiment in order to keep the results from our experiments compatible with one another.

#### Constructing the inner crowds

Inner crowds were compiled by aggregating across the first through tenth estimates, separately for each set size, within each individual.

#### Constructing the outer crowds

Outer crowds were generated by selecting each of our 63 participants and randomly grouping them with 9 other participants. This process gave us 63 crowds of 10 people. The estimates were aggregated by set size within each crowd; only first estimates from each set size were used in the outer crowds. The order in which estimates were added to the aggregate was randomly determined, with the constraint that participants would serve as the first guess in the one crowd for which they were systematically determined to belong to; this random order was consistent across set sizes for each crowd. (We chose to aggregate in a random order because there was no principled means of ordering participants; by contrast, for the inner crowd, responses were aggregated in chronological order.)

### Data analysis

Rather than calculate values of variance and bias directly from the data, as is done in Table 1, we instead estimated those values by fitting a mixed-effects nonlinear model to each crowd type and assessing the resulting parameter estimates. Specifically, we fit the parabolic function *a*/t + *b* to our observed values of MSE_P_ (see Rauhut and Lorenz 2011), where t is the number of estimates being aggregated (which is also the trial number for the inner crowd), *a* is the estimated variance of a set of responses, and *b* is the estimated bias (i.e., the asymptotic performance) of a set of responses. Note that in our analyses estimates of *a* and *b* are in terms of squared proportional deviance because that is the scale of our dependent variable, MSE_P_. Because crowd wisdom is equal to the variance of a crowd's responses, *a* also serves as an estimate of crowd wisdom (Page 2007). For both parameters, we included group-level effects for each participant (or outer crowd) and each set size. We furthermore allowed for a nested structure between set sizes and participants (or outer crowds) to reflect the fact that each set size was estimated multiple times by a given person (or outer crowd).

We fit these models using Bayesian parameter estimation in the "brms" package in R statistical software (Bürkner 2019). We placed a half-normal prior with a mean of 0 and a standard deviation of 0.5 on the *a* and *b* parameters. We used bounded priors because neither parameter would be interpretable if it were estimated to be negative.

We used Bayesian hypothesis testing to analyze the population-level parameter estimates from our model. Specifically, we obtained Bayes factors by calculating a Savage-Dickey ratio, which is the ratio of the zero-point-densities of the posterior and prior distributions for a given parameter (Wagenmakers et al. 2010). We will report Bayes factors in terms of the alternative hypothesis, BF_10_. We will consider evidence convincing when values are either 3 or greater (for the alternative) or 0.33 or less (for the null).

### Results

Before analyzing the data from Experiment 1, we removed all estimates that were at least one order of magnitude greater or smaller than the correct answer (e.g., an estimate of 5 or 500 when viewing 50 asterisks), because they seemed more likely to be a typo than a sincere estimate. Only 12 of 3780 estimates were removed.

**Fig. 2:**
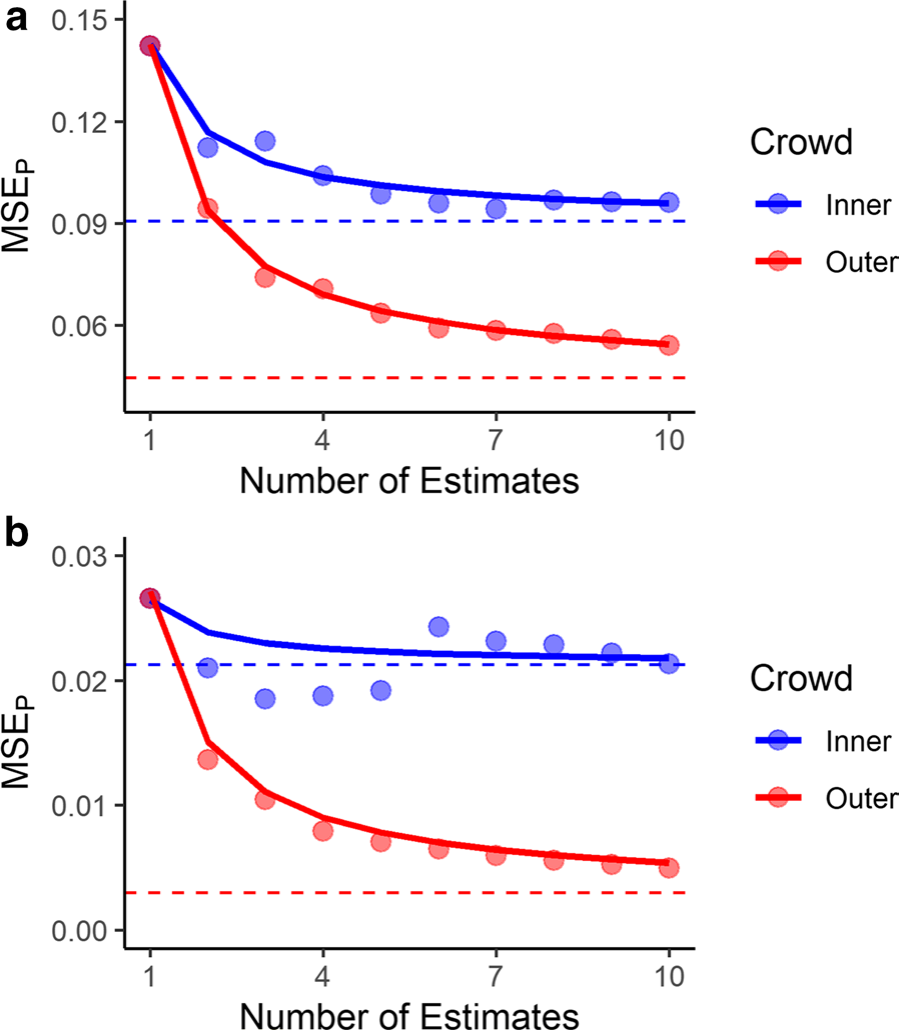
MSE_P_ of the inner (blue) and outer (red) crowds in Experiments 1 (**a**) and 2 (**b**) as a function of number of estimates. Note the different scales of the y-axes. The dots indicate MSE_P_ averaged over set size; lower MSE_P_ values indicate greater response accuracy. The solid lines indicate the population-level parabolic function, *a*/t + *b*, estimated from our mixed-effects models. The dashed lines indicate estimated crowd bias (i.e., *b*). The asymptotic advantage of estimating based on a crowd versus a single response (i.e., *a*) can be seen by comparing the dashed lines to the leftmost dot; this gap represents the effect size of crowd wisdom

The population-level parabolas estimated by our mixed-effects models are presented in Fig. 2a. Parameter estimates from the models are presented in Table 2. We found very strong evidence in favor of nonzero values of *a* and *b* for both the inner and outer crowd. These findings mean, respectively, that both crowd types benefitted from response aggregation and both crowd types were biased away from the true values being estimated.

**Table 2:** Means and standard deviations of posterior distributions from our two-parameter nonlinear model

Experiment	Crowd	Parameter	*M*	SD	BF_10_
1	Inner	*a*	0.05	0.01	1417.13
		*b*	0.09	0.01	9.83 × 10^14^
	Outer	*a*	0.10	0.02	9.78 × 10^14^
		*b*	0.045	0.005	1.69 × 10^90^
2	Inner	*a*	0.005	0.004	0.02
		*b*	0.021	0.003	4.60 × 10^16^
	Outer	*a*	0.024	0.004	7.28 × 10^14^
		*b*	0.003	0.001	5.14

Corresponding Bayes factors are presented in the rightmost column

These parameters correspond to the function *a*/t + *b*, in which *a* is estimated crowd variance (i.e., crowd wisdom) and *b* is estimated crowd bias. BF_10_ values ≥ 3 or ≤ .33 indicate convincing support for the alternative or null hypothesis, respectively

### Discussion

Experiment 1 demonstrated that the wisdom of the inner and outer crowd extended to our numerosity estimation task. To our knowledge, this experiment is the first demonstration of inner-crowd wisdom for numerosity estimation in the context of an experimentally controlled design (see van Dolder and van den Assem 2017, for an observational study). It appears that individuals rely on a process akin to sampling from an inner distribution when making numerosity judgments, thereby allowing those judgments to benefit from estimate aggregation—in this case, even without a long delay between estimates (see Vul and Pashler 2008).

## Experiment 2

In Experiment 2, we sought to extend our findings by assessing the impact of expertise on crowd wisdom. We did so by giving our participants a short set of training trials prior to beginning the critical trials. Numerosity estimation tends to become substantially more accurate after training (e.g., Izard and Dehaene 2008). However, training might also overly constrain the variance of estimates. This restricted variance could result in redundant information being added to the aggregate, in which case crowd wisdom would subsequently suffer.

### Methods

#### Preregistration

We preregistered Experiment 2 and followed the methodology outlined in the preregistration document. However, we decided to analyze our data in a different way than what is outlined in that document. (We did not preregister the method or analyses for Experiment 1.)

#### Participants

Participants were 62 people recruited from Amazon's Mechanical Turk service. These participants were selected in the same manner as for Experiment 1.

#### Procedure

We made one change to our experimental procedure for Experiment 2. Before beginning the 60 critical trials, participants now first completed a set of 12 practice trials in which they received corrective feedback after providing an estimate. These trials were comprised of set sizes ranging from 50 to 100 stars, just as would be the case for the subsequent critical trials. To prevent participants from knowing that they would only see six set sizes during the critical trials, we structured the practice trials so that the set sizes ranged from 50 to 100 stars in increments of five^4^; furthermore, the program randomly added or subtracted up to two stars from each set size. For example, a set of 60 asterisks was randomly altered to include 58–62 asterisks.

### Results

As in Experiment 1, we removed estimates that were at least one order of magnitude greater or smaller than the correct set size. Only 22 of 3720 estimates were removed.

The results are presented in Fig. 2b and Table 2. Unlike Experiment 1, we observed a strong null effect for *a* when estimating the inner crowd, indicating that after training, the inner crowd did not outperform individual responses. Additionally, individuals were still biased away from the true values based on the convincingly nonzero value for *b*. For the outer crowd, we obtained convincing evidence that both *a* and *b* were nonzero, suggesting that outer-crowd wisdom was still present after training and that this training did not eliminate the bias of the outer crowd.

### Discussion

Training was extremely effective in Experiment 2; the MSE_p_ of participants' first guesses was roughly five times smaller than it was in Experiment 1. For the inner crowd, however, this enhanced accuracy came at the expense of severely restricted variance; the null *a*-value estimated for the inner crowd suggests that an infinite number of estimates would not be more accurate than a single estimate. In contrast, aggregating across the outer crowd still enhanced accuracy.

## General discussion

In two experiments, we demonstrated that (a) inner- and outer-crowd wisdom extended to our novel numerosity estimation task and (b) training eliminated the wisdom of the inner crowd—that is, responses aggregated within individuals were not more accurate than unaggregated individual responses—while outer-crowd wisdom remained. To the extent that our experimental paradigm parallels the difference between novices and experts in other domains, our results suggest that novices benefit from both the inner and outer crowd, but that experts are better off consulting their fellow experts rather than attempting to aggregate multiple self-generated estimates.

More work is needed to determine how our findings generalize to novices and experts in tasks that are performed in daily life (see, for example, Dawes et al. 1989). One way to more naturally evaluate novices versus experts would be a paradigm in which participants may opt in to provide estimates. For example, Bennett et al. (2018) found that outer-crowd accuracy was enhanced by optional responding (i.e., allowing people to decide to respond to a general-knowledge question or not). They reasoned that people are aware of those questions for which they have the most relevant knowledge and that they will choose to answer those questions more often, leading to more accurate outer crowds than does forced responding. However, Bennett et al. (2018) did not formally assess differences in accuracy between single individuals and the crowd. To the extent that optional responding leads to a higher proportion of expert participants, our data suggest that crowd wisdom (and particularly inner-crowd wisdom) may suffer from optional responding.

Future work could examine whether learners are sensitive to the effect of expertise on the value of the inner crowd. Previous studies have looked at learners' decisions to average (or not) multiple self-generated responses (Müller-Trede 2011; Fraundorf and Benjamin 2014; Herzog and Hertwig 2014b). But whether peoples' decisions to average estimates are impacted by their expertise or by task difficulty has not been directly investigated.

Aggregating multiple judgments is a simple but extremely effective way of arriving at an accurate estimate. We have shown here that both the inner and outer crowd are beneficial for numerosity estimation when participants are novices, but that the enhanced performance yielded by training obviates the benefits of aggregating across the inner crowd.

## Data Availability

A preregistration document for Experiment 2, our complete data set, and an R script that replicates all analyses are available online at the Open Science Framework at https://osf.io/ja3xg/.

## Footnotes

1 We define crowd wisdom as the gain in accuracy afforded by the crowd over a single estimate (e.g., Rauhut and Lorenz 2011).

2 In both experiments, we wanted a sample size of 60 participants for an analysis that we ultimately abandoned. Even so, our samples provided sufficient power for the analyses that we present here.

3 Note that we did not compute a proportional bias for the crowd because that value was estimated from a nonlinear model, as described in the "Data analysis" section.

4 Note that this should have given us only 11 practice trials. A programming error resulted in one additional 50-star trial.
